# Managing eye health in young children

**Published:** 2010-03

**Authors:** Aderonke Baiyeroju, Richard Bowman, Clare Gilbert, David Taylor

**Affiliations:** Professor of Ophthalmology, College of Medicine, University of Ibadan, Nigeria.; Ophthalmologist and Director of Training, CCBRT Hospital, Dares Salaam, Tanzania; Honorary Senior Lecturer, London School of Hygiene and Tropical Medicine.; Co-director, International Centre for Eye Health, London School of Hygiene and Tropical Medicine; Clinical Advisor, Sightsavers, UK.; Chairman, International Council of Ophthalmology Examinations, International Council of Ophthalmology, 11–43 Bath Street, London EC1V 9EL. d.taylor@ich.ucl.ac.uk

**Figure:**
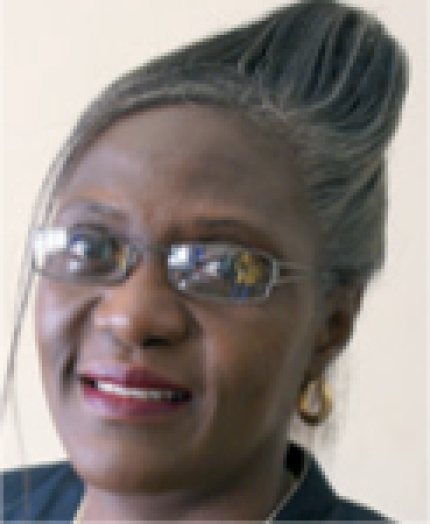


**Figure:**
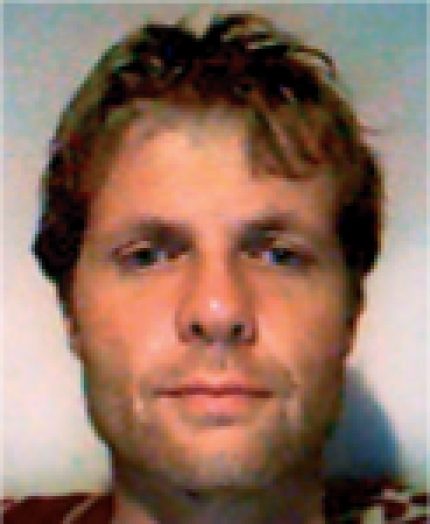


**Figure:**
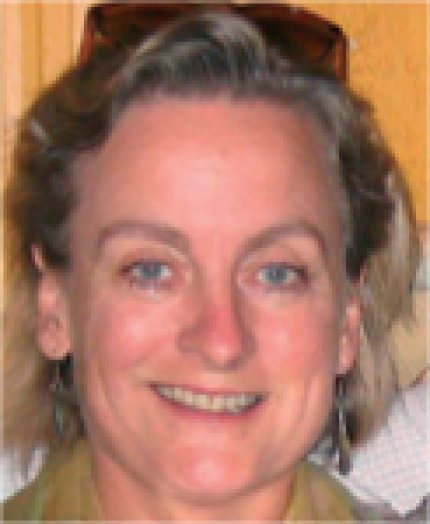


**Figure:**
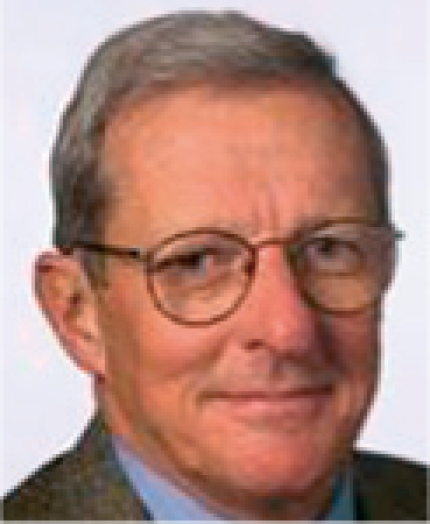


Children are brought to us with a range of conditions, usually when their parents or carers notice something is wrong. This article focuses on the more challenging complaints in babies and young children, who are the most difficult to assess. This is not an exhaustive list of presenting complaints or examination techniques, but it will give a starting point.

**Figure:**
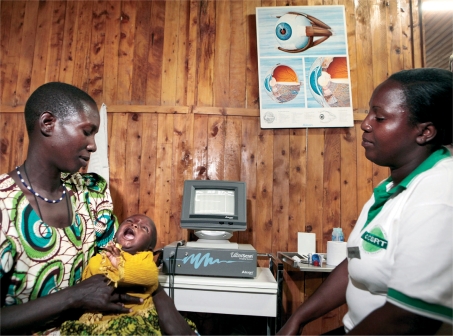
Always believe the mother. TANZANIA

CCBRT/Dieter Telemans

## General principles

When your patient is a young child:

- **Do the best you can, and start treatment or refer the child as quickly as possible.** The earlier the treatment starts, the better a child's vision is likely to be after treatment. Even if there is no treatment, a blind baby or child still needs help to develop as normally as possible and should also be referred.
- **Believe the parents.** Most things parents notice and mention to you are real and relevant. Parents are usually right! They spend a great deal of time with their children, and will observe how children behave and what their eyes look like.
- **Listen more than you speak.** Usually the parent will help you towards the diagnosis.
- **Don't take any chances - play it safe.** If in doubt, ask a colleague or refer the child to a specialist.
- **Be patient.** It takes time to let the parents tell their story and to examine a child properly, especially one who cannot or will not cooperate.
- **Plan ahead.** If you have a busy clinic, see any young children first. If you don't, they may get tired and irritable, which will cause stress for their parents or carers; it also makes children difficult to examine.

## Communication with parents

Good communication with parents is essential:

- **Speak in ways that parents can understand.** Speak in simple, everyday terms and use diagrams or drawings to support your explanations.
- **Be as honest as you can.** This could include saying that you are uncertain of what exactly is wrong.
- **Be kind.** Parents want what is best for their children, but because of lack of education or resources they may not always make the best choices. Do not blame parents for what they have done, or what they have not done. This may make them less likely to seek further help. With careful explanation, you can help them to make the best decision for their child's eyes and vision.

## Referral

When you refer a child, it is very useful to write a referral letter. Give the letter to the parents to take with them and keep a copy for your records. In the letter, state:

- what the mother complained of or noticed
- what you found when you examined the child
- what you have done, if anything (e.g., started antibiotics).

It is important to encourage parents to take up a referral.

- **Explain why you might refer the child.** If a child needs to be referred, it is very important to convince the parents that specialist testing and treatment will help their child.
- **Help parents to understand the urgency of seeking further help.** Tell them how important it is to get the advice. However, don't alarm parents unnecessarily. Explain that the quicker a child gets treatment, the better the outcome will be.
- **Be supportive.** Advise parents about what support is available to help them take up the referral, such as transport, subsidies, and so on. If you can, tell them what to expect at the hospital and what they should bring with them (such as the referral letter, clothes, or food).

Always refer children with the following eye problems urgently:

- One or both eyes are abnormally small or large (Figure 1)
- One or both eyes stick out (Figure 2)
- There is a red mark on the eyelid (Figure 3)
- One or both eyes are obviously abnormal; for example, white all over (Figure 4).

## Assessing vision in a baby (0–1 year)

Don't be anxious about examining a baby. If the baby is awake and attentive, there is a lot you can find out by asking the parents and simply observing the baby's reactions.

- First ask the parents what they think about their baby's vision.
- Notice how the baby looks at things in the room, such as the window or any lights.
- Watch for eye contact between the baby and parents.
- Does the baby look when someone comes into the room?
- Does the baby respond to silent smiles or to raised eyebrows?
- Do you get eye contact?

**Figure:**
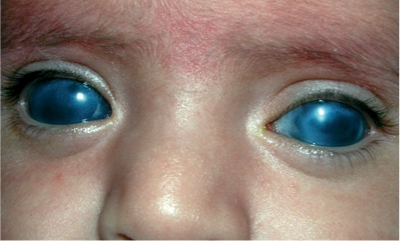
Figure 1. Both eyes of this child are enlarged and the corneas are cloudy. The child should be referred for urgent investigation

David Taylor

**Figure:**
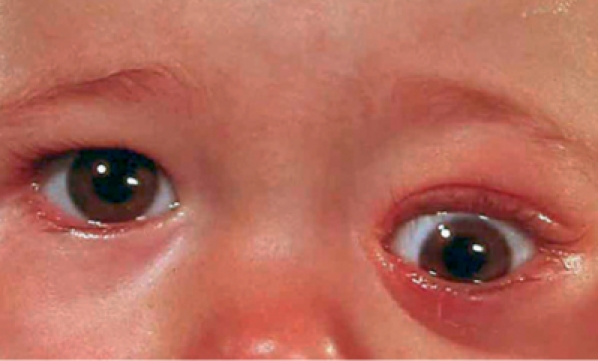
Figure 2. The left eye of this child is protruding and displaced downwards. The child should be referred for urgent investigation

David Taylor

**Figure:**
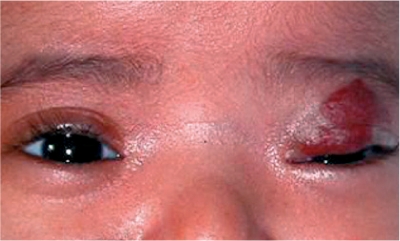
Figure 3. A red mark on the eyelid may be a haemangioma, which can cause amblyopia (lazy eye). The child should be referred for urgent investigation

David Taylor

**Figure:**
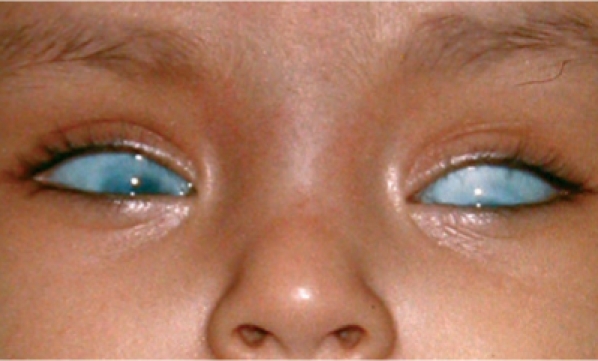
Figure 4. Both eyes are obviously small and white. The child should be referred for urgent investigation

David Taylor

You should have realistic expectations about what a baby should be able to do by a certain age. Table 1 shows when a baby is too young to show a visual response, when the response is likely to develop, and at what age you should be worried if a baby does NOT show the expected response. You can ask the mother or check the baby's responses yourself.

For example, if a baby of about three weeks old does not turn to a diffuse light, such as light coming from a window, you would not necessarily be worried - although you would still believe the parents if they are concerned. On the other hand, if a baby is eight weeks old and does not eventually turn to a diffuse light, then there may be a problem and you should investigate further.

Bear in mind that there can be a lot of variation in babies’ development; however, this table should be a helpful guide.

**Figure:**
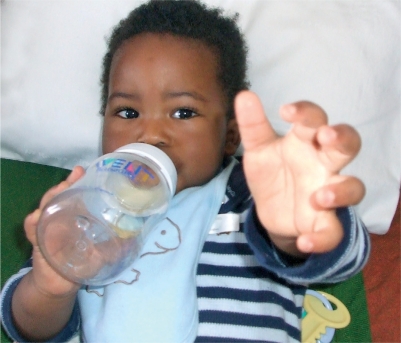
Figure 5. A healthy baby with good fixation. It is clear that he can see the camera and is reaching out for it.

Winjiku Mathenge

**Figure:**
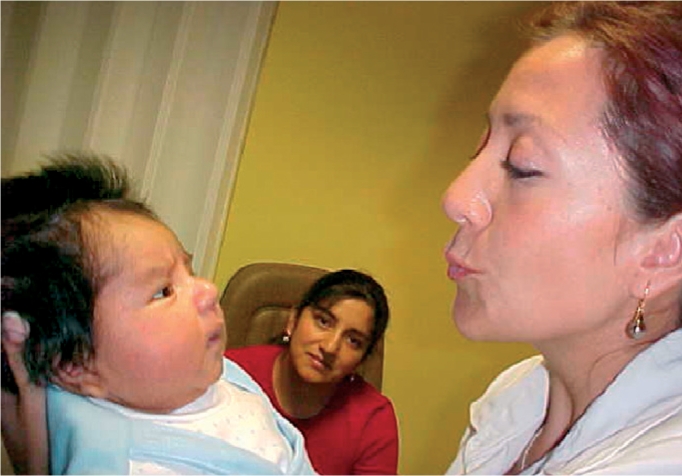
Figure 6. An eye care worker checks a baby's fixation. The baby is looking at her face, which is a reassuring sign.

Aderonke Baiyeroju

**Table 1:** Normal visual functioning for a baby

**Behaviour**	**Age**				
	**Neonate**	6 weeks	3 months	4 months	**5 months +**
Blinks when a light is flashed in their eyes?	Healthy babies will do this. If not, suspect a problem				
Turns to a diffuse light, such a light coming from a window?	May do it	Healthy babies will do this. If not, suspect a problem			
Looks at your face when 10–20 cm away (less than 1 foot)? Any response to silent smiles or eyebrow raising?	Too young	May do it	Healthy babies will do this. If not, suspect a problem		
Eyes fix on, and follow, a dangling ball or toy?	Too young	May do it	Healthy babies will do this. If not, suspect a problem		
Watches an adult at 1.5 metres (5 feet)?	Too young	May do it		Healthy babies will do this. If not, suspect a problem	
Converges accurately? (If you move a toy closer and further away, do the eyes focus on the toy and line up properly?)	Too young	May do it		Healthy babies will do this. If not, suspect a problem	
Blinks in response to a threat? (Any silent, sudden movement close to the face which causes no breeze, e.g., opening your fist very suddenly.)	Too young	Too young	Too young	May do it	Healthy babies will do this. If not, suspect a problem

**Figure:**
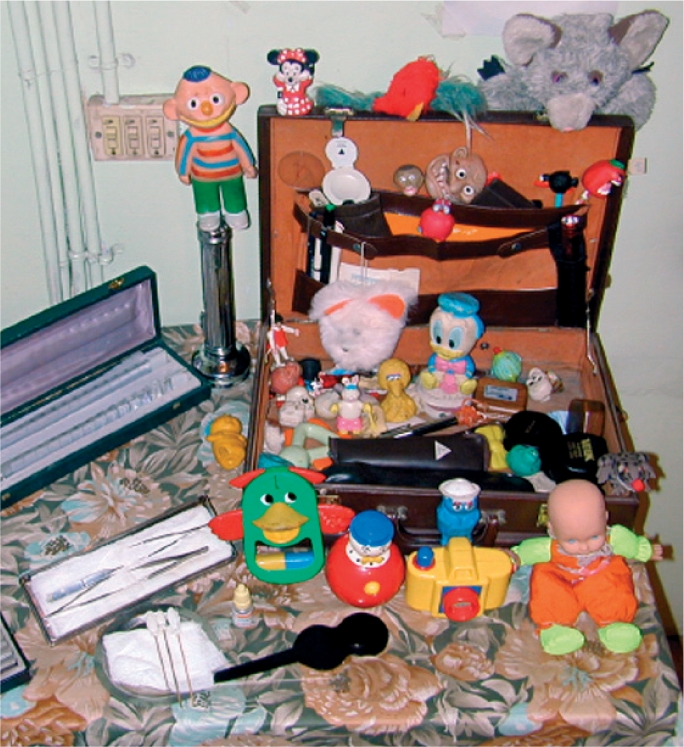
Figure 7. A lot of toys are needed to attract children's attention and to make examination fun!

Clare Gilbert

### Tips for examining a baby

- Try to carry out as much of the examination as possible without touching the baby. Children often resist having their eyes held open, for example.
- Have many toys available (Figure 7). For each new toy, the baby will momentarily hold their eyes steady, allowing a quick examination. If available, use toys which are bright and can flash on and off. A good rule to remember is **one toy, one look.**
- Don't be embarrassed about making funny noises! These help to attract the baby's attention and to keep them interested and calm.
- In order to be able to do a more detailed examination in an infant, examine the child while he or she is being bottle fed or breast fed.
- If you are struggling, ask the parent's permission to wrap the baby. Place the baby on a blanket or sheet, hold the arms to the side and the legs straight, and wrap the blanket around the body and arms (Figure 8). Ask the parent to hold the baby. Either the parent or a helper can then carefully open one eye at a time for the examination (without putting pressure on the eye - see Figure 2 on page 17). Remember that this may be very stressful for both the baby and the parent.

**Figure:**
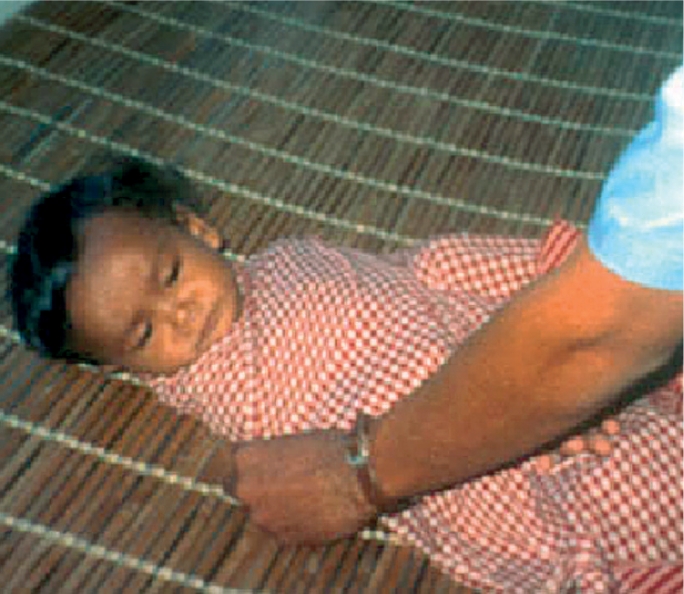
Figure 8. Wrapping a baby for an eye examination

ICEH

**Figure:**
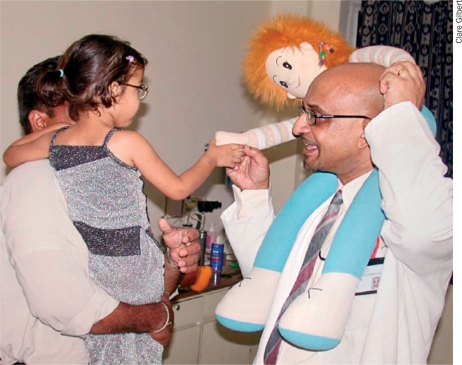
Figure 9. Be playful and make a game of the examination

Clare Gilbert

## Assessing vision in a young child (1–5 years)

Children in this age group should have steady eyes, no squint, no history of sight difficulties and, if in a good mood, show interest in colourful or interesting objects in the room. They should respond to silent smiles, eyebrow raising, and winking.

Children in this age group should also be able to see objects presented in their peripheral visual field by a colleague while you draw their attention to your face, perhaps by making a funny noise. Cover one eye at a time if the child will allow it and ask them to identify different sized objects or, with older children, letters - make it a game.

Many children can accurately name colours by the age of three years but many cannot until they are older; it is reassuring if they can.

After the age of three, most children can participate in accurate visual acuity, visual field, and colour vision testing by someone trained and with age-appropriate equipment.

If you do not have that equipment or have not been trained to use it, you can still test a child's functional vision using everyday objects as described above.

### Tips for examining a young child

The tips for examining a baby (above) apply equally well to young children. In addition:

- Be playful and make a game of the examination (Figure 9). For example, shine a light into the mother's eye first, or pretend you are playing ‘hide and seek’ or ‘peekaboo’ when covering one eye.
- Observe children when they don't know they are being observed, for example while you are talking to the mother or taking a history.
- The tip about wrapping up a baby will work for a younger child, but may be more difficult in an older child. Ask the parents what they think would be appropriate or would work best. For example, parents may prefer to hold their child's arms gently.

**How to use this article**

The rest of this article is divided into four sections, each of which is based on what the mother or parents will say when they bring their child to see you:

1. “My child cannot see”
2. “There is something white in my child's eye(s)”
3. “My child's eyes are wobbly” or “My child has a squint”
4. “My child's eyes are red and/or sticky”

For each problem, the article describes the likely causes, what you should ask the parents, what you should look for, what action you should take, and how you can talk to the parents. Where appropriate, these are described separately for babies and young children. We hope you find this useful!

## 1 “My child cannot see”

**Table:** 

**Possible causes**	**Further possible causes: babies**	**Further possible causes: young children**
Corneal scar/opacity Cataract Glaucoma Developmental problems (retina, optic nerve, brain)	Retinal conditions such as meningitis and retinopathy of prematurity (ROP), which is rare in Africa Central nervous system conditions, e.g. following prolonged or difficult birth	Retinal conditions, such as retinal dystrophies, CMV retinitis (a complication of HIV), late presentation of ROP Central nervous system conditions, e.g. following meningitis, malaria, or head injuries
**What to ask the parents**	**Additional questions: babies**	**Additional questions: young children**
When did you first suspect there was something wrong with your child's vision? Does your child dislike bright light? If yes, suspect glaucoma or some form of retinal dystrophy. Does the eye water? If yes, this may simply be a blocked nasolacrimal duct, in which case the eye will probably also be sticky. However, if the watering happens when the child is in bright light, or if the child also cannot see or is in pain, you should suspect congenital glaucoma (Figure 10). Does the baby seem to be in pain? If yes, it may be glaucoma or there may be a problem with the cornea. Was the baby premature and cared for in a neonatal unit? If yes, it may be ROP Was the birth of the baby difficult or long? If yes, it may be cerebral visual impairment.	Is there a history of fever? If yes, suspect neonatal meningitis.	Is there a history of head injury or fever immediately before the difficulty with vision was noticed? If yes, suspect a central nervous system condition. Can the child walk around and hear normally? If no, suspect a central nervous system condition. Do the parents or brothers and sisters have (similar) vision problems? If yes, suspect an inherited retinal problem or an environmental problem such as maternal ingestion of drugs or alcohol.
**What to look for**	**What else to look for: babies**	**What else to look for: young children**
Use a torch to examine the cornea. Is there a corneal ulcer or scar/opacity? How big is it? Is the pupil completely covered? Check the lens in each eye. Use a torch to look just behind the pupil. A cataract will appear white. Do the red reflex test (see box on page 11). A cataract blocks the red reflex, so it will appear black or partially black (Figure 11).	Assess the visual milestones given in Table 1, page 5.	Assess the child's vision using the tips on page 6. **Hint:** In this age group, doing a red reflex test is often easier because you can turn it into a game. For instance, tell a child of two or three years old to ‘blow the light out’ (you switch it off!). You don't need to get very near: 30 cm (about 1 foot) will do, as long as the light is bright. Practise on an older brother or sister, or the mother, first; this reassures the child that the test is not frightening.

**Figure:**
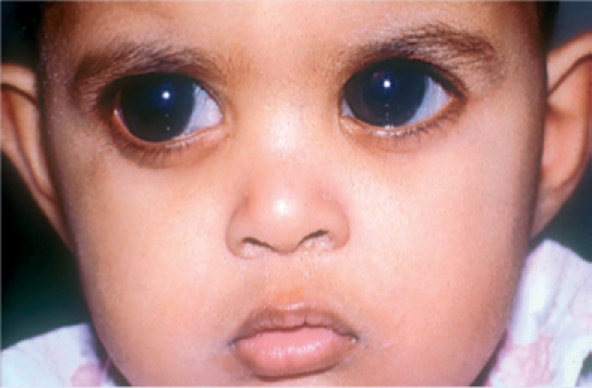
Figure 10. Congenital glaucoma

P Khaw

**Figure:**
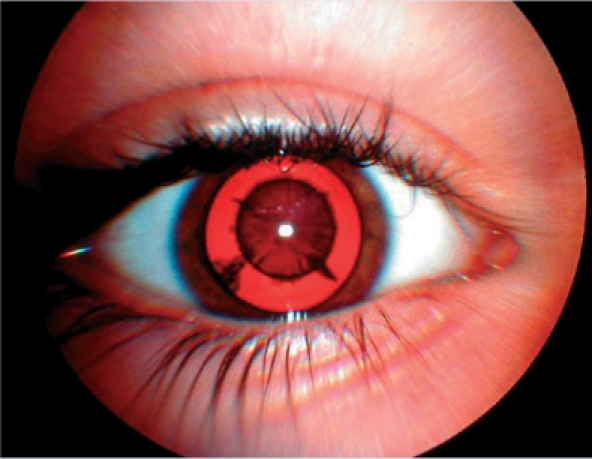
Figure 11. The pupil was dilated using one drop of cyclopentolate 0.5%. The cataract is visible as a black shadow obstructing the red reflex.

David Taylor

### What to do

- Always refer babies or young children who have something obviously wrong with their eyes and/or vision
- Always refer when you and/or the parents are concerned about the baby's vision and when you think their vision may be outside the normal for their age.
- Remember to err on the side of caution-always believe the mother. If you are unsure, it is better to refer than to miss something serious.
- When a baby needs a referral, refer him or her to an ophthalmologist, preferably one trained in paediatric ophthalmology, whatever the suspected cause.

**Remember:** Cataracts in children are not the same as cataracts in adults. Children with visual loss from cataracts need **urgent** surgical treatment to prevent them from developing amblyopia (lazy eyes) which may not be reversible if surgery is delayed. They should **not** be told to “wait for the cataract to mature”; nor to “come back when your child is older”. These messages can cause delays in treatment which can have a lasting impact on the child.

### What to tell parents when you refer their child

- It is important to persuade parents to take up their baby's referral urgently - just as soon as they can. The sooner the exact nature of the condition is known, the sooner they can be treated and the better the outcome is likely to be.
- Say something like this: “It's difficult for me to find out exactly what is wrong and/or how much your child can see - your child may need more tests. Knowing exactly what is wrong will help us find out whether your child's condition can be treated.”
- Try to dissuade parents from seeking the advice of traditional healers or using traditional remedies. These may be harmful and may delay much-needed investigation and treatment.

## 2 “There's something white in my child's eye(s)”

**Table:** 

**Possible causes**	**Further possible causes: babies**	**Further possible causes: young children**
**On the surface of the eye:** corneal ulcer (Figure 12) or corneal scar/opacity (Figure 13) which may cover the pupil. **Just inside the eye:** cataracts (Figure 14), which can cause a white pupil. **At the back of the eye:** retinoblastoma (Figure 16), coloboma (Figure 17), ROP (unlikely in Africa). These can also cause a white pupil, but the whiteness comes from deeper inside the eye.	A white spot on the surface of the eye can be due to congenital abnormalities and is often bilateral. Corneal scars, ulcers, or opacities can be due to ophthalmia neonatorum (usually bilateral), trauma, or the use of harmful traditional remedies.	Corneal ulcer or scar/opacity is usually due to harmful traditional remedies or measles and vitamin A deficiency. At the back of the eye, additional causes can include CMV retinitis (a complication of HIV), late presentation of ROP, or other developmental abnormalities; all are serious.
**What to ask the parents**		**Additional questions: young children**
When did you first notice it? Is it both eyes or just one eye? Most of these causes can affect one or both eyes. When do you see it? All the time, or just when the light is coming from any particular direction - such as over your shoulder when you are feeding your baby or cuddling your child? If all the time, it's likely to be due to corneal opacity or cataract, if only some of the time, then it could be cataract, retinoblastoma or coloboma. Was your baby premature and cared for in a neonatal unit? If yes, it could be ROP or late presentation of ROP Have you used any treatment or traditional remedies?		Did the child have a fever, rash, or diarrhoea before the white spot developed? If yes, this could indicate corneal ulcer or scarring as a result of measles or vitamin A deficiency.

**Figure:**
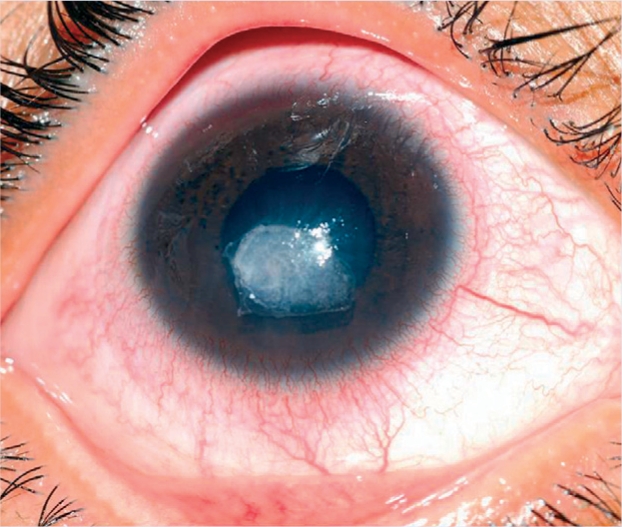
Figure 12. Corneal ulcer with circumcorneal congestion

P Vijayalakshmi

**Figure:**
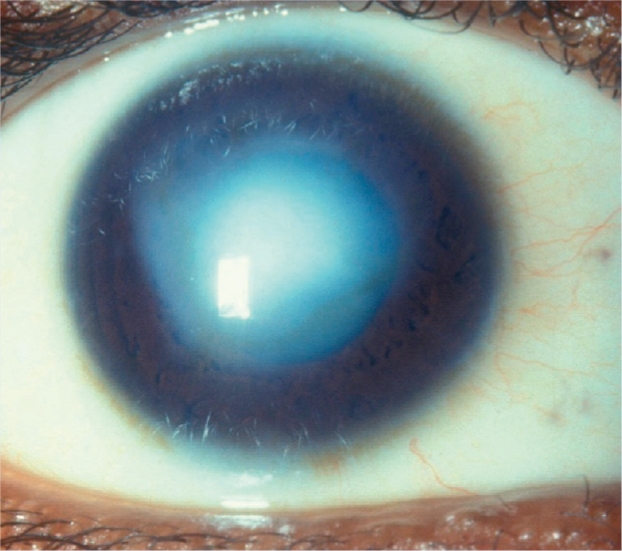
Figure 13. Corneal scar/opacity

Clare Gilbert

**Figure:**
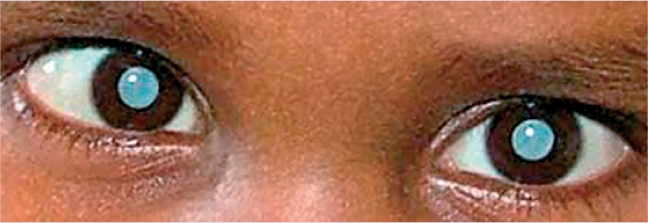
Figure 14. Bilateral cataract

ICEH

**Figure:**
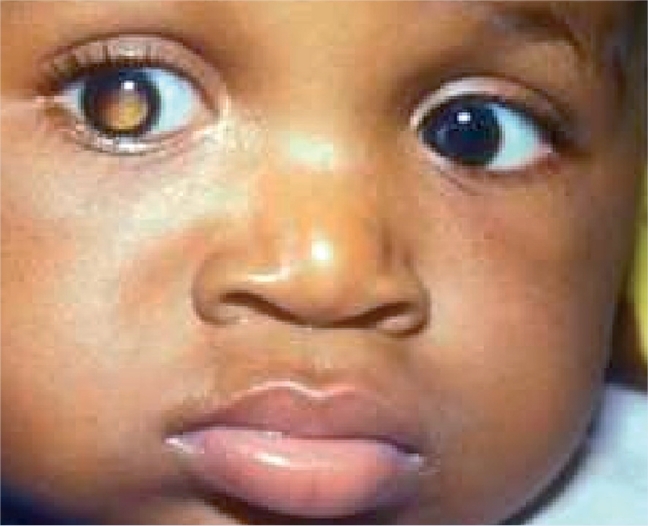
Figure 15. Retinoblastoma presenting as a white reflex. It can also present as a squint, or with loss of vision (if bilateral).

Pak Sang Lee

**Figure:**
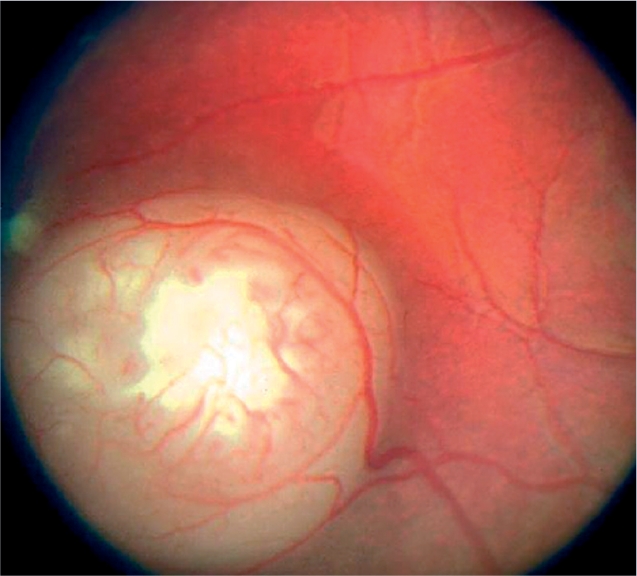
Figure 16. Retinoblastoma as seen with a direct ophthalmoscope. Can present as a white reflex or a squint, or with loss of vision (if bilateral)

ICEH

**Figure:**
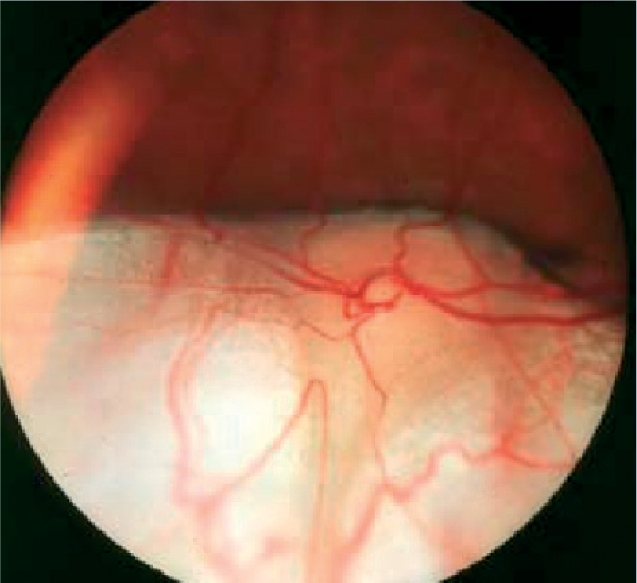
Figure 17. Chorioretinal coloboma as seen with a direct ophthalmoscope. Can give a white reflex in young children

ICEH

### What to look for

#### On the surface of the eye

- Use a torch to examine the cornea. Is there a corneal ulcer or scar/opacity? How big is it? Is the pupil completely covered?
- Are there Bitot's spots (Figure 18)? If yes, this is an indication of vitamin A deficiency.

#### Just inside the eye

- Check the lens in both eyes using a torch. A cataract will appear white.
- Do the red reflex test. A cataract blocks the red reflex, so it will appear black or partially black (Figure 5).

#### At the back of the eye

- Do the red reflex test. A white reflex is abnormal and could be retinoblastoma/coloboma or another problem. If you have dilating drops, dilate the pupils and examine with a direct ophthalmoscope.

**Hint:** Babies and young children can be difficult to examine and you may not be able to see a white reflex, particularly if the reflex is coming from the back of the eye. Parents often see the white reflex more easily than you do because they are with the child more and they are likely to see the eyes in different lighting conditions - such as when they are looking at the child with the light coming over their shoulder. That is just one reason why it is important to always believe the parents!

### What to do

- **If there is an ulcer,** start a topical antibiotic immediately, show the parents how to instil the antibiotic (every 30 minutes), and refer very urgently. For babies, you may need two people to instil the antibiotic - one to hold the baby and the other to instil the drops (see article on page 17).
- **In older children with corneal ulcers,** this may be due to vitamin A deficiency, in particular if Bitot's spots are also present. Give a dose of 200,000 international units (IU) immediately if the child is over 12 months of age and also start a topical antibiotic. Refer.
- Refer all children with **suspected corneal scarring/opacity** so vision can be assessed and they can be examined to see whether treatment is possible.
- Refer all children when there is an obvious white reflex just inside the eye(s) or deeper in the eye(s).
- Refer all children whose parents say they have seen something white in the eye - **even if you can't see** it. It's really important not to miss a retinoblastoma - if diagnosed and treated early, this can save a child's sight and their life. Err on the side of caution: refer the child with a letter explaining what you have seen or what the parents have reported and urge parents to take them to the hospital **within two days.**
- Refer all cases to an ophthalmologist, preferably one trained in paediatric ophthalmology.

**Figure:**
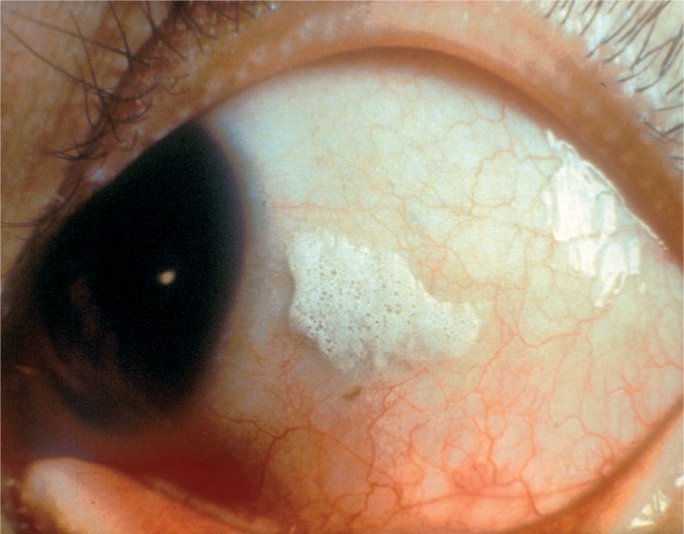
Figure 18. Bitot's spots are an indication of Vitamin A deficiency. Note the typical white, foamy appearance on the surface of the conjunctiva, next to the iris.

ICEH

### What to tell parents when you refer their child

- Try and dissuade parents from seeking the advice of traditional healers or using traditional remedies. These may be harmful, but just as important, they may cause a delay in the proper investigation and treatment of children.
- If you suspect an **ulcer,** explain that parents must put the eye drops in every 30 minutes until they reach the hospital. They must be urged to go immediately - **no delay.** Explain that it is important to find out the exact cause of the ulcer so that it can be treated properly; the antibiotic eye drops are just an emergency treatment.
- If you can see something white **just inside or at the back of the eye,** say something like: “I agree with you that there does seem to be something white inside the eye. To find out exactly what the condition is and what the right course of treatment would be, your child needs to be seen by a trained ophthalmologist who has more equipment than I do. It is important to go within two days.”
- If you cannot see something white in the eye, say something like: “Even though I cannot see anything today, I believe you and you did the right thing to bring your child for examination. To find out exactly what the condition is and what the right course of treatment would be, your child needs to be seen by a trained ophthalmologist who has more equipment than I do. It is important to go within two days.”

### Implications beyond the clinic

- If measles is the underlying cause of a corneal problem, you need to be aware that more children may be affected. You should alert the agency responsible for immunisation.
- If you suspect vitamin A deficiency, be aware that there is likely to be more children affected in the community.
- If traditional remedies have been used, health education is important.

## 3 “My child's eyes are wobbly” or “My child has a squint”

### Possible causes

There are two main causes of wobbly eyes (nystagmus) and squint (where the eyes are misaligned):

1. Any condition which causes **loss of vision** may result in wobbly eyes or squint. If the loss of vision is in both eyes, the eyes can become wobbly; if the loss is in one eye, it can lead to squint.
2. An **abnormality in the brain mechanisms or muscles** which control the movement and position of the eyes can also lead to wobbly eyes or squint, even if the eyes themselves are entirely normal.

### What to ask the parents

- When did the parents first notice the condition?
- Do the parents think their child can see normally?
- Does the squint point inwards or outwards?
- Have the parents noticed any other abnormality in one or both eyes, such as a white pupil?

### What to look for

- **Are the eyes straight and steady most of the time?** Before six weeks, many children's eyes wander from time to time. This is entirely normal. After six weeks the eyes should be basically steady and point in the same direction most of the time. There should some eye contact when your face is near theirs.
- **Check the vision.** If you cover each eye in turn with your or the mother's hand, does the baby object to you covering one eye in particular? The child might move their head or try to remove your hand. If this happens, the eye not being covered may have poor vision.
- Check for any **obvious abnormality** in one or both eyes, including something white in the eye (see above).
- Do a red reflex test (see page 11).
- Check pupil reactions.
- Which eye is turning?

### What to do

- Refer any children with wobbly eyes or a definite squint, especially babies with recent squint or eyes that point outwards. A squint may be the first sign of a more serious condition, such as retinoblastoma.
- Refer to an ophthalmologist, preferably one trained in paediatric ophthalmology.
- In all cases, refer to the hospital with a letter saying what you have seen. Make sure that the parents know that they need to be seen within a month.
- In some communities, a squint is seen as attractive, particularly in girls. However, it is important that parents realise that a squint may be a sign of something more serious.

**Figure:**
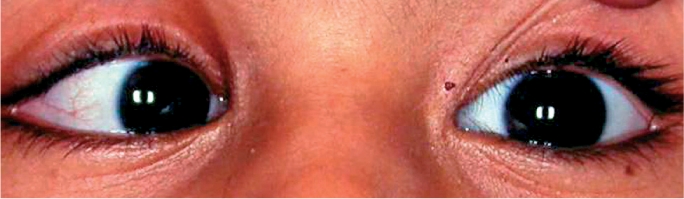
Figure 19. A child with squint. The right eye is turned inwards

David Taylor

### What to tell parents when you refer their child

- Tell parents that there may be something wrong with their child's eye and/or vision. Their child needs further examination and may be helped by treatment.
- Urge parents to take up the referral within one month.

For more information on squint, see article on page 12.

## 4 “My child's eyes are red and/or sticky”

**Table:** 

**Possible causes**	**Further possible causes: babies**	**Further possible causes: young children**
Viral, bacterial or fungal conjunctivitis Corneal ulcers Traditional eye remedies Foreign bodies Trauma	**Ophthalmia neonatorum.** This is infective keratoconjunctivitis starting within 28 days of birth.	**Allergic conjunctivitis.** This can occur at any age but is unusual in infancy. **Vernal keratoconjunctivitis** (spring catarrh). This is unusual below three years of age but can occur in older children. It is usually bilateral. **Trachoma.** This can occur at any age but is more common in young children.
**What to ask the parents**	**Additional questions: babies**	**Additional questions: young children**
How old is the child? If under 28 days, suspect ophthalmia neonatorum. When did the redness and stickiness start? Is there a history of trauma or eye injury? Ask the parents exactly what happened. Have traditional eye remedies been used?	Does the mother or father have a urogenital infection? If yes, suspect ophthalmia neonatorum.	Does anyone else in the family or community have the same problem? If yes, suspect vernal conjunctivitis or trachoma. Does the child have any other problems - itchy skin rash or wheezing? If yes, suspect allergic conjunctivitis.
**What to look for**	**What else to look for: babies**	**What else to look for: young children**
Is the discharge watery or thick and yellow? Thick and yellow discharge is likely due to bacteria, including Gonococcus. If it is watery, this may be due to viral conjunctivitis or a corneal ulcer. Can you see a corneal ulcer? This might be due to an injury that became infected, traditional eye remedies, or infection with Gonococcus or another organism. Examine the eyes carefully for signs of injury. Evert the eyelids to look for foreign bodies. Are both eyes affected, or just one eye?	If the child is under 28 days old, the eyelids are swollen, and the discharge is thick and yellow, it is most likely due to ophthalmia neonatorum.	Evert the upper eyelids. ‘Cobblestones’ (Figure 22) are a sign of **vernal conjunctivitis.** The eyes are usually irritable with a watery, stringy discharge. Follicles and/or intensive inflammation (Figure 23) on the inner surface of the upper eyelids are likely due to **trachoma.** Active trachoma will often be irritating and have a watery discharge. If the eyes are itchy, watery, and red it could be **allergic** conjunctivitis. The conjunctiva may also be swollen. If the child is over 28 days old, there is no ulcer, and the eyes are watery and red, this could be **viral or bacterial conjunctivitis** - especially if the eyes are sticky.

**Figure:**
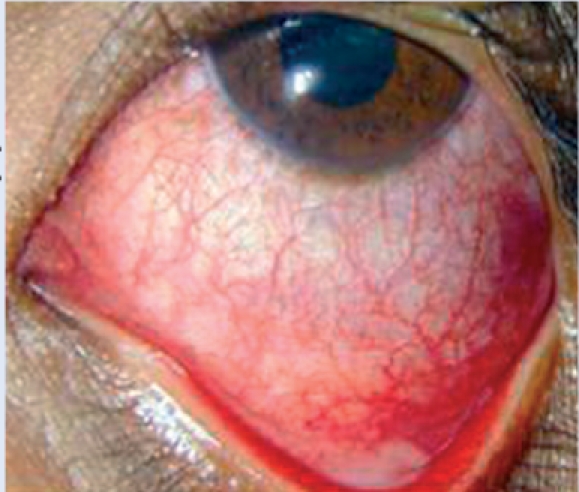
Figure 20. Bacterial conjunctivitis. The eyes are inflamed and there is a purulent discharge. It is usually bilateral. If unilateral, it may follow mild trauma or be due to a foreign body in the eye.

P Vijayalakshmi

**Figure:**
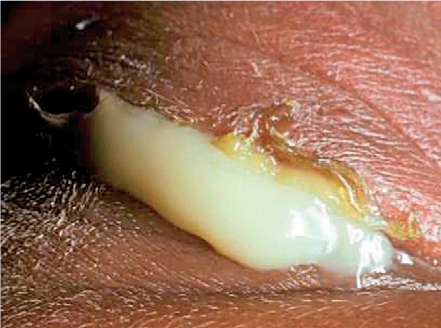
Figure 21. Ophthalmia neonatorum due to **Gonococcus** infection. The lids are swollen and there is copious discharge. The eye is in serious, immediate danger

Murray McGavin

**Figure:**
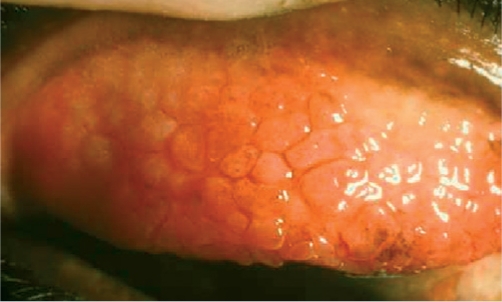
Figure 22. ‘Cobblestones’ typical of vernal keratoconjunctivitis

ICEH

**Figure:**
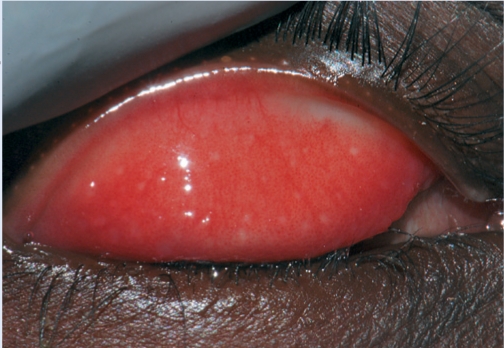
Figure 23. Follicles and inflammation typical of trachoma

Emma Harding-Esch

### What to do

- If you suspect **ophthalmia neonatorum,** start treatment immediately - clean the eyelids and instil topical antibiotics. Show parents how to clean the eyelids and instil antibiotic eye drops. Then refer urgently - tell parents to continue eye drops until the child is seen. Systemic antibiotics are also needed.
- **If there is an ulcer,** start a topical antibiotic immediately, show the parents how to instil the antibiotic (every 30 minutes), and refer urgently.
- **Suspected viral/bacterial conjunctivitis:** Start a topical antibiotic (repeated every two hours) and follow up in two to three days. Show the parent or carer how to instil the eye drops (see page 17).
- **Allergic and vernal conjunctivitis** can be treated with sodium chromoglycate drops or topical antihistamine drops, if available. Children with severe vernal conjunctivitis will need more aggressive treatment and should be referred to an ophthalmologist.
- **Trachoma:** The child should be treated with one dose of systemic azithromycin. If unavailable, use topical tetracycline eye ointment which will have to be applied twice a day for six weeks.
- **Burns:** If a chemical or other fluid entered the eye, wash the eyes as shown in ‘How to irrigate the eye’ (Vol 18 No 55, see Useful Resources on page 11) and refer immediately.
- **Foreign body:** Carefully remove it with the edge of a clean, folded cloth or a matchstick covered in cotton wool. Refer if embedded.
- **Blunt injury:** Advise rest. Refer children with hyphaema (blood in the anterior chamber) if it looks severe or has not improved after three days of rest. Aspirin should be avoided.
- **Penetrating injury:** Refer urgently.
- **If the child is in pain,** analgesics (paracetamol or ibuprofen) may be given. Avoid the use of aspirin.
- **For any injury,** the most important thing is to give frequent antibiotic drops and make sure that the child is taken to an ophthalmologist as soon as possible.

### What to tell parents when you refer their child

- If you think the baby might have **ophthalmia neonatorum,** the baby and both parents need to be investigated and treated. Delay in treatment can permanently damage the child's sight.
- If you suspect an **ulcer,** explain that parents must put the eye drops in every 30 minutes until they reach the hospital. They must be urged to go immediately - **no delay.** Explain that it is important to find out the exact cause of the ulcer so that it can be treated properly; the antibiotic eye drops are just an emergency treatment.
- Suspected **viral, bacterial, allergic,** and **vernal conjunctivitis:** Tell parents that the infection should get better, but that you want to see the child again in a few days to make sure there is improvement. Even if the eye or eyes get better quickly, parents should still bring the child back so you can see them again, because there may be incomplete healing or there may be some damage that still needs treatment.
- Whatever the cause of the redness or discharge, tell parents to avoid using traditional remedies or seeking the advice of a traditional healer.
- Explain that it is really important to instil eye drops as often as instructed, and in the correct way.
- In case of a suspected penetrating injury, explain that an ophthalmologist needs to see the child urgently and parents should continue with the antibiotics until the child is seen.
- In case of a blunt injury, parents should come back if the eye does not settle within a few days of the injury. The child may need a referral.

### Implications beyond the clinic

If a child presents with trachoma, it is almost certain that other children and adults living in the same village or community will also have trachoma. Unless the whole community is treated, the child will be re-infected. Record the community the child is from and alert the people responsible for trachoma control in your district.

**How to see the red reflex**

The red reflex test can reveal problems in the cornea, lens, and sometimes the vitreous. It can alert you to large lesions in the retina but it cannot be used to identify causes of poor vision related to retinal or optic nerve damage, such as retinal dystrophy or optic nerve hypoplasia.

- The red reflex is much easier to see in a darkened room, so switch off the lights, draw the curtains or ask the parents to accompany you into a room which doesn't have a window.
- Use a direct ophthalmoscope or a red reflex scope (both of these devices allow you to look directly down the light beam) and make sure the batteries are well charged!
- Stand between one and two feet away (around one third to two thirds of a metre) and direct the light to one eye at a time: you should see a bright red reflex from the pupil.
- Sometimes the reflex is more pink than red. This is when the light beam is directed towards the optic disc which is normally pink, not red like the retina (see Figure 24). It is useful to practice looking for the pink reflex. With a co-operative patient, ask them to look slightly away from the light, for example at one of your ears (your left ear if you're examining their left eye, and your right ear if you're examining their right eye). Move nearer and further away until you can spot the pink reflex.

**Figure:**
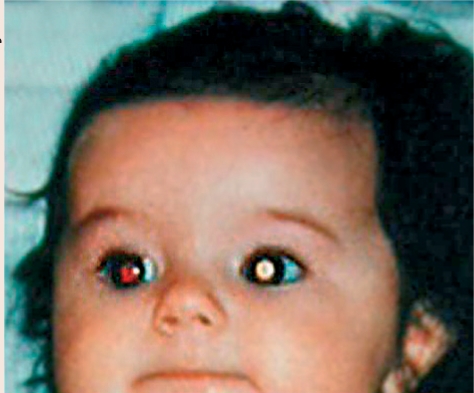
Figure 24. A normal red reflex (right eye) and a normal pink reflex (left eye)

David Taylor

## Conclusion

You can have a significant impact on reducing visual loss and blindness in children by examining and referring them. Remember, one of your most powerful tools is your good communication with the parents. By helping parents to understand the importance of a referral and supporting them to take up that referral quickly, you can improve the chances of a good visual outcome for their child.

Even if you suspect that there's nothing that can be done to help the eye or vision, there is still a lot you can do to help the child and parents. In addition to referring the child to an ophthalmologist, make sure the family receives all the other services it may need, including support for the parents and low vision care, rehabilitation, and visual stimulation for the child.

